# Universal pandemic precautions—An idea ripe for the times

**DOI:** 10.1017/ice.2020.327

**Published:** 2020-07-03

**Authors:** David J. Weber, Hilary Babcock, Mary K. Hayden, Sharon B. Wright, A. Rekha Murthy, Judith Guzman-Cottrill, Sarah Haessler, Clare Rock, Trevor Van Schooneveld, Corey A. Forde, Latania K. Logan, Anurag Malani, David K. Henderson, for the SHEA Board of Trustees

In the early 1980s, the human immunodeficiency virus (HIV) epidemic in the United States led to a paradigm shift in infection prevention. Published papers reported that healthcare personnel (HCP) were at risk for acquisition of HIV and hepatitis B virus (HBV) via percutaneous, mucous membrane, or non-intact skin exposure. The number of acute HBV infections among healthcare personnel (HCP) was estimated as 17,000 infections in 1983.^1^ In the United States, 58 confirmed and 150 possible cases of occupationally acquired HIV infection were reported to the Centers for Disease Control and Prevention (CDC) between 1985 and 2013.^2^ The threat of bloodborne pathogens to HCP led to institution of universal precautions (now called standard precautions), which recommend that HCP wear gloves for anticipated contact with all body fluids except sweat. Implementation of standard precautions plus pre-exposure hepatitis B vaccine, postexposure prophylaxis for HIV and HBV, and engineering controls (eg, blunted suture needles, self-sheathing needles, needleless connectors, etc) have dramatically reduced the risks for HIV and HBV acquisition by HCP. Since 1999, only 1 confirmed case of HIV (a laboratory technician who sustained a needle puncture while working with a live HIV culture in 2008) has been reported.^2^ The rate of HBV infections among HCP decreased ~98% from 1983 to 2010.^1^ Although some initial pushback occurred with the implementation of standard precautions due to concern that routine wearing of gloves would be poorly received by patients and impair the ability to perform procedures such as placing intravenous catheters, they are routinely practiced and accepted today.

Similarly, the threat proffered by the sudden appearance of coronavirus disease 2019 (COVID-19) in the healthcare workplace resulted in the implementation of risk mitigation strategies that may also produce permanent behavioral modification in the healthcare setting. As it also occurred during the 1980s with the HIV/AIDS epidemic, the introduction of a new disease and new risks into the healthcare setting should result in long-lasting changes in patient care that at least offer potential for increased patient and staff safety.

## Universal Pandemic Precautions

As of June 19, 2020, the COVID-19 pandemic has led to 2,201,399 infections and 118.458 deaths in the United States. The CDC has reported that HCP account for ~11% of COVID-19 infections.^3^ Increasing evidence has provided a roadmap for protecting HCP from the acquisition of COVID-19. Systematic reviews and meta-analyses have demonstrated the effectiveness of masks^4,5^ and eye protection^5^ to protect HCP. As with HIV and HBV, multiple measures need to be implemented in healthcare facilities to prevent SARS-CoV-2 transmission, including (1) screening of patients, visitors, and HCP for symptoms of COVID-19 prior to entry; (2) routine use of source control masks by patients, visitors, and HCP^6^; (3) frequent hand hygiene and surface disinfection of shared equipment and devices; (4) enhanced personal protective equipment for HCP performing aerosol-generating procedures and during care of known or suspected patients with COVID-19; and (5) prompt testing of persons with signs or symptoms of COVID-19 and institution of appropriate isolation precautions. Screening of asymptomatic patients at the time of admission may be of benefit in several circumstances, among them: (1) testing prior to admission to a congregate setting (eg, psychiatry, rehabilitation, post-surgery recovery units); (2) testing prior to admission to the labor and delivery unit to protect the nursery; and, (3) testing to identify presymptomatic patients because they are clearly infectious. Testing may also be useful for screening HCP who have had COVID-19 exposure as defined by CDC. Testing strategies are changing rapidly, and increased testing of asymptomatic patients and staff may be more practical in the near future, depending on infection prevalence and test availability.^7^

To protect HCP, patients and visitors from acquisition of COVID-19 and other respiratory pathogens, we propose a paradigm shift; implementation of universal pandemic precautions (UPPs): *Use of a mask and eye protection for all direct patient contacts or at a minimum, use of a mask and eye protection for direct patient contact when the patient is unable (eg, children) or unwilling to wear a mask*. We propose this paradigm shift based on the following: (1) transmission of SARS-CoV-2 has been well described from presymptomatic and likely occurs from asymptomatic COVID-19 patients; (2) A negative COVID-19 test in an asymptomatic patient does not exclude incubating COVID-19, and such patients may become infectious shortly after the test; (3) the use of masks^4,5^ and eye protection^5^ by HCP protects against acquisition of SARS and SARS-CoV-2; and (4) the use of universal pandemic precautions would prevent HCP from an exposure that, per the CDC, would lead to exclusion from work for 14 days.^8^ We also suggest that the implementation of universal pandemic precautions will likely also offer a potential beneficial effect on the prevention of transmission of other droplet-spread respiratory pathogens in the healthcare setting (eg, influenza A and B, respiratory syncytial virus, seasonal coronaviruses, etc), especially during seasons in which these and other respiratory viruses are circulating.

We understand that implementation of universal pandemic precautions will require careful messaging for our colleagues and patients. We expect that the rationale for aforementioned universal pandemic precautions will persuade our colleagues to accept them. Messaging to patients should focus on describing the rationale for universal pandemic precautions, including noting that their use is one of several precautions that protect patients. In addition, patients should be informed that this routine practice is not specifically focused on them individually. Finally, we realize that institution and discontinuation of universal pandemic precautions should be based on current local case numbers (or rates or burden) and local prevalence of infection in asymptomatic populations (eg, preprocedural test positivity rates because symptom screening cannot detect these potentially infectious patients.

We realize that paradigm shifts are difficult and, as demonstrated by Semmelweis,^9^ may not be accepted. Ultimately, however, the use of hand hygiene as advocated by Semmelweis became a keystone of infection prevention. We believe that universal pandemic precautions will be accepted by patients and staff and that it will ultimately result in a safer healthcare environment for all.
